# Deep Learning for Differentiating Benign From Malignant Parotid Lesions on MR Images

**DOI:** 10.3389/fonc.2021.632104

**Published:** 2021-06-23

**Authors:** Xianwu Xia, Bin Feng, Jiazhou Wang, Qianjin Hua, Yide Yang, Liang Sheng, Yonghua Mou, Weigang Hu

**Affiliations:** ^1^ Department of Radiation Oncology, Fudan University Shanghai Cancer Center, Shanghai, China; ^2^ Department of Oncology, Shanghai Medical College, Fudan University, Shanghai, China; ^3^ Department of Oncology Intervention, The Affiliated Municipal Hospital of Taizhou University, Taizhou, China; ^4^ Department of Infectious Disease, The Affiliated Municipal Hospital of Taizhou University, Taizhou, China; ^5^ Department of Radiology, The Affiliated Municipal Hospital of Taizhou University, Taizhou, China; ^6^ Department of Hepatobiliary Surgery, The Affiliated Municipal Hospital of Taizhou University, Taizhou, China

**Keywords:** MR image, parotid gland tumor, deep learning, classification, image processing

## Abstract

**Purpose/Objectives(s):**

Salivary gland tumors are a rare, histologically heterogeneous group of tumors. The distinction between malignant and benign tumors of the parotid gland is clinically important. This study aims to develop and evaluate a deep-learning network for diagnosing parotid gland tumors *via* the deep learning of MR images.

**Materials/Methods:**

Two hundred thirty-three patients with parotid gland tumors were enrolled in this study. Histology results were available for all tumors. All patients underwent MRI scans, including T1-weighted, CE-T1-weighted and T2-weighted imaging series. The parotid glands and tumors were segmented on all three MR image series by a radiologist with 10 years of clinical experience. A total of 3791 parotid gland region images were cropped from the MR images. A label (pleomorphic adenoma and Warthin tumor, malignant tumor or free of tumor), which was based on histology results, was assigned to each image. To train the deep-learning model, these data were randomly divided into a training dataset (90%, comprising 3035 MR images from 212 patients: 714 pleomorphic adenoma images, 558 Warthin tumor images, 861 malignant tumor images, and 902 images free of tumor) and a validation dataset (10%, comprising 275 images from 21 patients: 57 pleomorphic adenoma images, 36 Warthin tumor images, 93 malignant tumor images, and 89 images free of tumor). A modified ResNet model was developed to classify these images. The input images were resized to 224x224 pixels, including four channels (T1-weighted tumor images only, T2-weighted tumor images only, CE-T1-weighted tumor images only and parotid gland images). Random image flipping and contrast adjustment were used for data enhancement. The model was trained for 1200 epochs with a learning rate of 1e-6, and the Adam optimizer was implemented. It took approximately 2 hours to complete the whole training procedure. The whole program was developed with PyTorch (version 1.2).

**Results:**

The model accuracy with the training dataset was 92.94% (95% CI [0.91, 0.93]). The micro-AUC was 0.98. The experimental results showed that the accuracy of the final algorithm in the diagnosis and staging of parotid cancer was 82.18% (95% CI [0.77, 0.86]). The micro-AUC was 0.93.

**Conclusion:**

The proposed model may be used to assist clinicians in the diagnosis of parotid tumors. However, future larger-scale multicenter studies are required for full validation.

## Introduction

Parotid gland tumors are rare tumors, accounting for approximately 5% of head and neck tumors, and approximately 75% of them are benign. The most common types of parotid gland benign tumors are pleomorphic adenomas and Warthin tumors (1, 2).

The preoperative diagnosis of benign and malignant tumors of the parotid gland is of great clinical significance and can have an important impact on surgical planning. The choice of surgical procedure depends on the histological type of the tumor. Approximately 5% to 10% of pleomorphic adenomas have a risk of malignant transformation and a high risk of recurrence, and radical surgery is usually used to treat them. The malignant transformation of Warthin tumors is extremely rare, occurring for only 0.3% of patients. Tumor removal or conservative observation is recommended in clinical practice to avoid the risk of facial nerve injury due to surgery (3). Malignant tumors of the parotid gland request extensive resection (4).

It is difficult to diagnose malignant tumors of the parotid gland *via* clinical manifestations (5). Fine-needle aspiration cytology (FNAC) is often used for the preoperative diagnosis of parotid gland tumors, in which the accuracy in discriminating benign and malignant diseases is 87.8%-97% (6, 7). However, due to the difficulty of sampling and the heterogeneity of the tumor, fine-needle aspiration cytology is sometimes uncertain and not representative of the true nature of the tumor. It may also lead to the spread of tumor cells, increasing the possibility of local recurrence and sometimes increasing the risk of infection (8). Therefore, preoperative imaging plays an important role in evaluating the location and nature of the tumor for the surgical plan (9–11). Ultrasound and CT are common imaging methods for diagnosing parotid gland tumor (12). However, an inflammatory lump is not easily distinguishable from a tumor on the ultrasound image, and when the difference between the density of the tumor and the density of the parotid tissue was small, the clear boundary could not be obtained by general CT (12–14). MRI is also an important method for the diagnosis of benign and malignant tumors of the parotid gland due to the high resolution of soft tissues. The sensitivity of MRI for parotid gland tumors is 86%, and the specificity is 90% (15). However, because parotid gland tumors are relatively rare and the tumors are heterogeneous, there are obvious differences in the judgment of benign and malignant parotid gland tumors.

In recent years, with the development of artificial intelligence, the application of deep learning in the medical field has made rapid progress. Deep learning uses simple neurons to form a complex neural network (15). For medical image diagnostic assistance, deep-learning methods can outperform many traditional machine learning methods. Antropova et al. (16) extracted mammograms, ultrasound and MRI features and used them for deep-learning training combined with traditional computer-aid diagnosis (CAD) methods to develop systems that are superior to the traditional CAD analysis of single images. Wang et al. (17) used pretraining and transfer learning methods to fine-tune the network model for other classification tasks in predicting benign and malignant prostate cancer (PCa) on MR images. Yang et al. used multiparameter magnetic resonance imaging (mp-MRI) to diagnose and detect prostate cancer through a CNN and SVM cotrained model (18). These studies demonstrate that deep learning, especially of convolutional neural networks (CNNs), is superior to non–deep-learning methods.

In terms of practical application, many studies have shown that the performance of artificial intelligence or deep learning can reach or exceed that of physicians (19–23). Several studies have shown that deep-learning techniques are comparable to radiologists’ detection and segmentation tasks in MRI examinations (24). In a recent report, Zhao et al. (25) developed a deep-learning autoLNDS (lymph node detection and segmentation) model based on mp-MRI. The model can detect and segment LNs (lymph nodes) quickly, yield good clinical efficiency and reduce the difference among physicians with different levels of experience. However, the application of deep learning in medical image diagnosis is limited (26). Wang et al.’s (27) research shows that incorporating diagnostic features into neural networks is a promising direction for future study. Ma et al. (28) used complementary patch-based CNNs to extract low-level and high-level features and fused the feature maps to performing classification. This kind of operation may yield information from important feature domains, but networks based on single imaging series still have limitations. Therefore, this study aims to propose a multiseries-input CNN to boost the performance of MR image classification tasks.

At present, there is no relevant research based on neural networks to predict the type of parotid gland tumor (15). This is mainly due to the rarity of parotid gland tumors, as the incidence of common cancers is higher than that of parotid gland cancer (29), which therefore lack sufficient data. On the other hand, due to the rarity of and limited clinical experience with parotid gland tumors, the significance of their auxiliary diagnosis will be substantial. The distinction between malignant and benign tumors of the parotid gland is clinically important.

This study aims to develop a system to act as an intelligent assistant in medical image diagnosis based on deep-learning technology and to design a model for predicting parotid gland tumors.

## Methods and Materials

### Workflow Introduction

**Figure 1:**
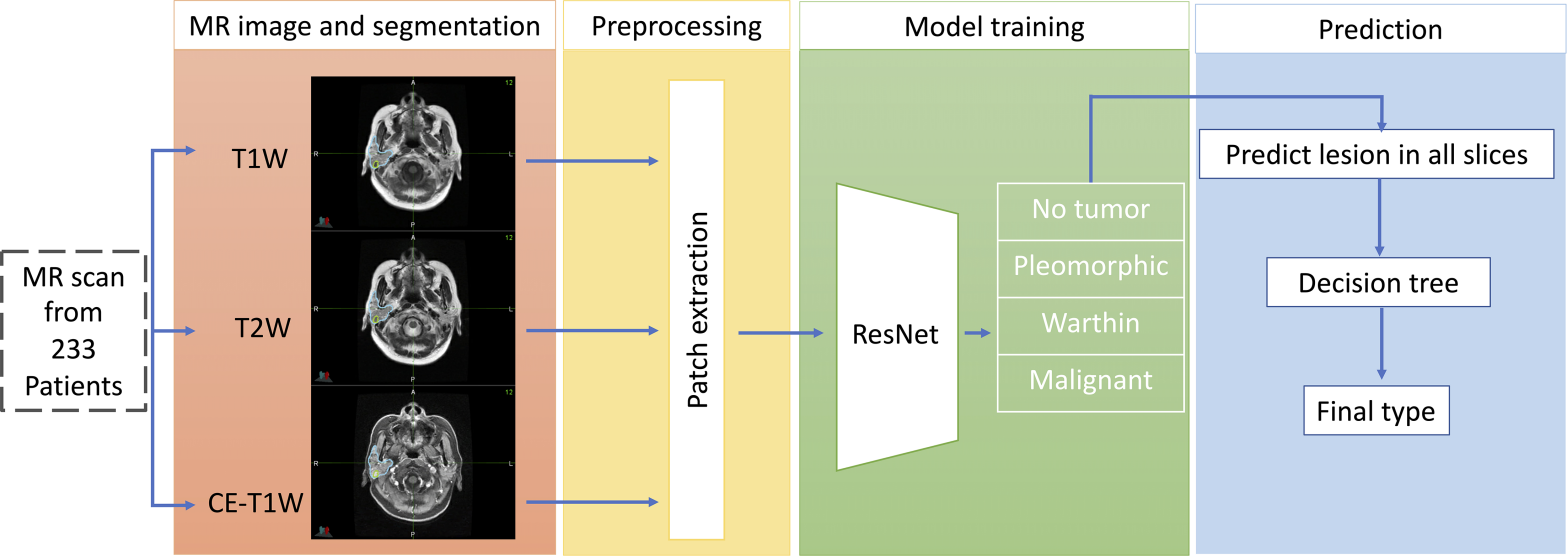
The workflow of our proposed deep-learning framework for the differentiation of benign from malignant parotid lesions. The first part shows multimodal MR images and tumor segmentation. The second part shows the preprocessing stage for the MR images. The third part shows the training network prediction model and tumor type classification. The final part comprehensively shows that predictions are made for all slices to determine the tumor type.

**Figure 1** shows the whole workflow of our research. The MR images of 233 patients were collected. The tumor and parotid gland were segmented manually by physicians. A modified ResNet18 model was used to discriminate different parotid lesions.

#### Patients and MR Image Acquisition

Two hundred thirty-three patients were enrolled in our study (**Table 1**). All patients were treated from 2014-2018 at Fudan University Shanghai Cancer Center. There were 159 males and 74 females with an average age of 52.4 (range, 21-93 years). The histopathology results were acquired by the operation, and each tumor have only one histology result from patient’s pathology report. Patient pathology information was collected from the EMR system.

**Table 1:** Patient characters.

		Characteristics
Age		52.4 (21~93) years
Sex	Male	159 (68%)
	Female	74 (32%)
Pathology Type	Warthin tumor	63 (27.0%)
	Pleomorphic adenoma	90 (38.6%)
	Adenocarcinoma	80 (34.3%)
Site	Left	101 (43.3%)
	Right	114 (48.9%)
	Both	18 (7.7%)

This study focuses on two types of benign tumors (pleomorphic adenoma and Warthin tumor) and one type of malignant tumors (adenocarcinoma). The MRI scan parameters were based on our parotid gland MR scanning protocol and fine-tuned during scanning by the MRI operator. The details of the imaging parameters are shown in **Table 2**. All patients were scanned with at least three series (T1-weighted, T2-weighted and contrast-enhanced T1-weighted).

**Table 2:** MR scan parameters.

		Signa HDxt (GE)	Verio (SIEMENS)	Skyra (SIEMENS)
Patients		166 (71.2%)	34 (14.6%)	33 (14.1%)
T1-weighted	TR (Repetition Time)	280~540 ms	450~620 ms	250~1560 ms
	TE (Echo Time)	8.5~10.4 ms	12~16 ms	2.5 ~12 ms
T2-weighted	TR (Repetition Time)	2740~3600 ms	2500~5240 ms	2500~5790 ms
	TE (Echo Time)	84~88 ms	78~91 ms	78~83 ms
contrast-enhanced T1-weighted	TR (Repetition Time)	175~280 ms	4.1~6.0 ms	3.7~6.0 ms
	TE (Echo Time)	1.8~3.4 ms	1.5~2.5 ms	1.4~2.4 ms
	Contrast Agent	Gadopentetic acid	Gadopentetic acid	Gadopentetic acid
Slice Thickness		5~7 mm	4.5~7.2 mm	4.0~6.0 mm
Pixel size		0.4~0.6 mm	0.65~0.97 mm	0.4~0.85 mm

As a reference, 215 patients’ contralateral normal parotid glands (991 slice images in total) Were selected as negative samples.

#### Parotid Gland and ROI Delineation

**Figure 2:**
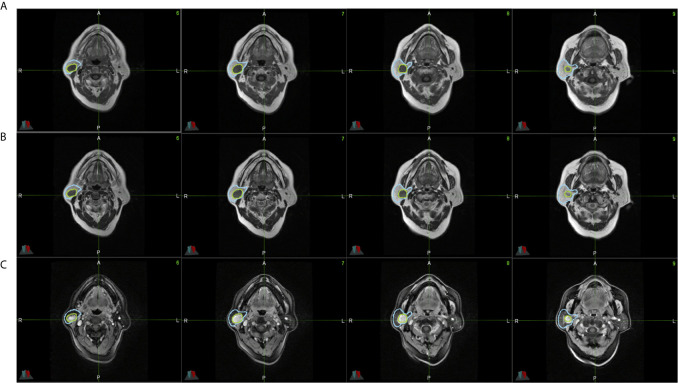
All parotid gland and tumor ROIs from a single patient’s lesion on MR images. **(A)** shows T1-weighted MR images. **(B)** shows CE-T1-weighted MR images. **(C)** shows T2-weighted MR images. The blue region is the parotid gland, and the green region is the tumor.

The parotid gland tumors were segmented by a radiologist with 10 years of clinical experience based on the MR series. The delineation tasks were performed on MIM (version 6.8.10, Cleveland, US). These contours were double-checked by a physicist. To improve the performance of the deep-learning model, the entire parotid gland was also segmented. **Figure 2** shows an example of this delineation.

#### Dataset and MR Image Preprocessing

After tumor and parotid gland segmentation, 3791 parotid gland region images were cropped from the MR images. A label (pleomorphic adenoma, Warthin tumor, malignant tumor or free of tumor), which was based on histological results, was assigned to each image.

**Figure 3:**
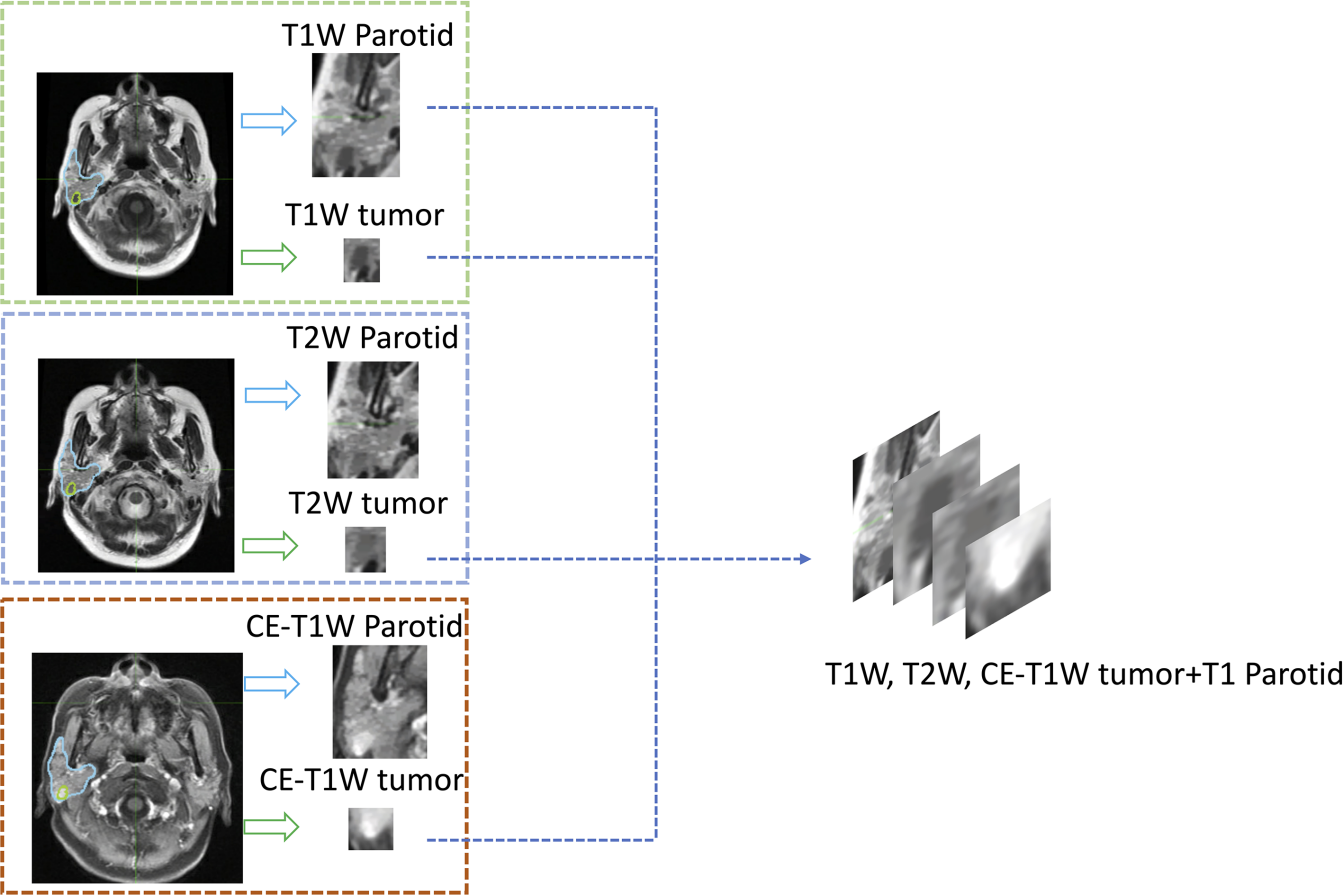
An example of the four-channel input. All parotid glands and tumors were cropped from segmented MR images, and then the three series of tumor images and the T1-weighted parotid gland image were input into different channels of one image.

To train the deep-learning model, these data were randomly divided into training and test sets at a ratio of 9:1, with 212 patients in the training set and 21 patients in the test set. The training set included 73 adenocarcinoma, 83 pleomorphic adenoma and 58 Warthin tumor patients, 2133 total slices with lesions (861 adenocarcinoma slices, 714 pleomorphic adenoma slices, and 558 Warthin tumor slices), and 902 total slices without lesions. The test set included 8 adenocarcinoma, 8 pleomorphic adenoma and 5 Warthin tumor patients, 186 total slices with lesions (93 adenocarcinoma, 57 pleomorphic adenoma, and 36 Warthin tumor slices), and 89 slices without lesions. Details on the processing workflow for the MR images are shown in **Figure 3**. A four-channel image was generated as the model input; the first three channels consisted of T1-weighted, CE-T1-weighted, and T2-weighted MR images, and the fourth channel included images of the parotid glands, which were contoured by a radiotherapist. A total of 991 parotid gland images without lesions were used as negative samples.

#### Deep-Learning Network Structure

**Figure 4:**
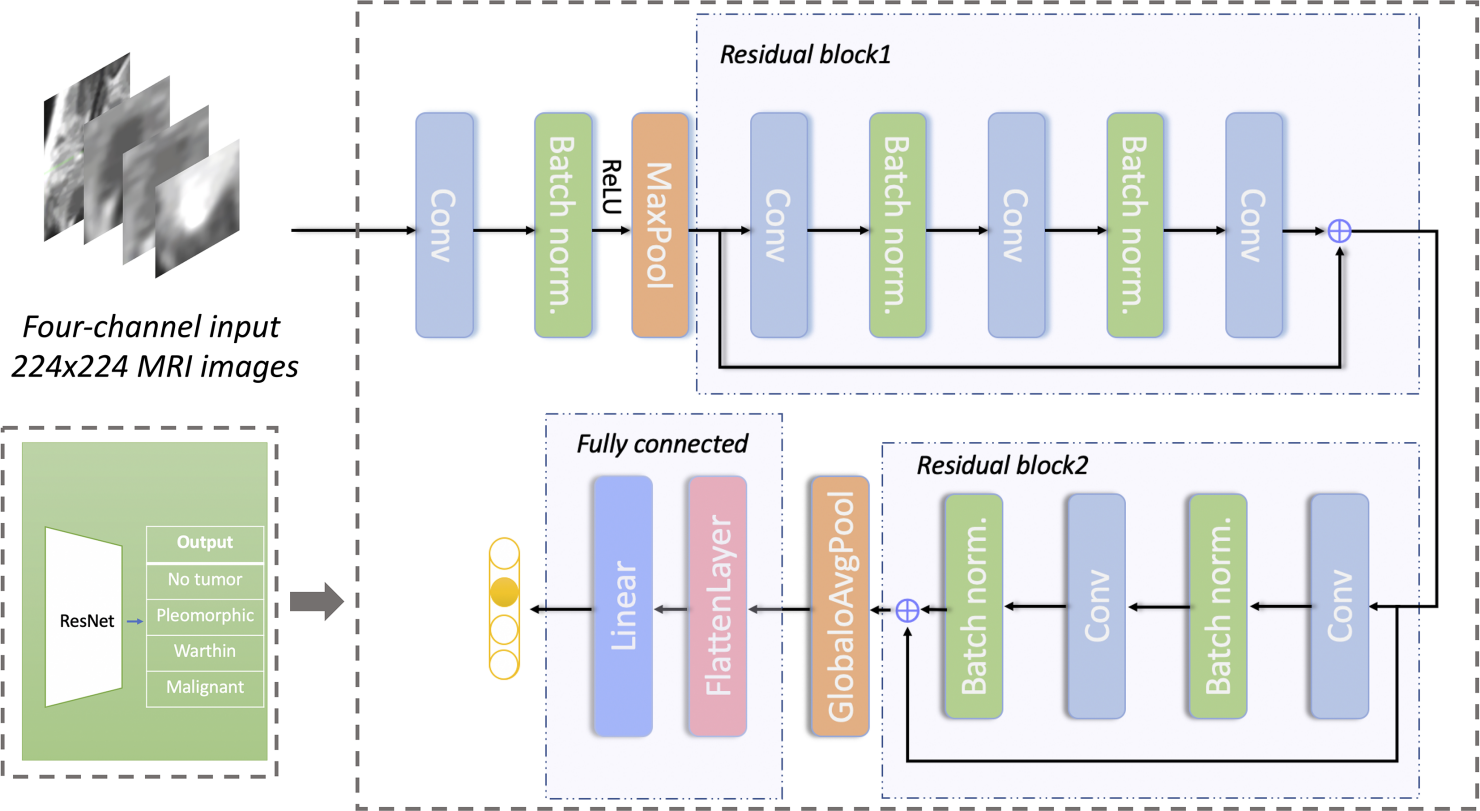
Network structure for predicting different types of parotid gland tumors based on ResNet. The network has 2 residual blocks. Conv, convolutional layer; Batch norm, batch normalization; Maxpool, max-pooling layer; GlobaloAvgpool, global average pooling layer; Linear, linear layer.

The detailed prediction network structure is shown in **Figure 4**. The network architecture is based on ResNet18. The input images were resized to 224*224 pixels. Random image flipping, contrast adjustment, color jitter, and affine transform were used for data augmentation. All image pixel values were normalized to [0, 1]. The batch size of training set and test set was 32. To avoid overfitting, we reduced the number of network layers (30) (i.e., the number of residual blocks were reduced to 2) and adjusted the number of network layers appropriately. The cross-entropy loss function (31) and the Adam optimizer (32) were used. The model was trained for 1200 epochs; the learning rate was 1e-6 for the first 600 epochs and was then multiplied by a factor of 0.8 every 100 epochs. An Intel I7-8700K CPU and Nvidia GeForce 1080 Ti GPU were used for model training. It took approximately 2 hours to complete the whole training procedure. The program was developed with PyTorch (version 1.2).

#### Performance Evaluation

The model performance was reported as its accuracy, which for binary classification was computed by:

$$\text{Accuracy}=\frac{\text{TP}+\text{TN}}{\text{Total}}$$

where *TP* denotes the number of true positives, *TN* denotes the number of true negatives, and *Total* represents the number of total samples. For multiclassification, the calculation of accuracy was computed by:

$$\text{Accuracy}=\frac{\text{TP}}{\text{Total}}$$

$$\text{Total}=\text{TP}+\sum_{\text{n}=1}^{\text{i}}\text{F}{\text{P}}_{\text{i}}$$

where FP_i_ represents the number of false negatives of the ith-class negative sample.

### The Impact of Different Image Series

To investigate the impact of different image series on the prediction accuracy, we filtered the input to the model using only a specific sequence of input models. Each dataset was trained separately until the accuracy of the model converged.

### Automatic Patient Diagnosis

**Figure 5:**
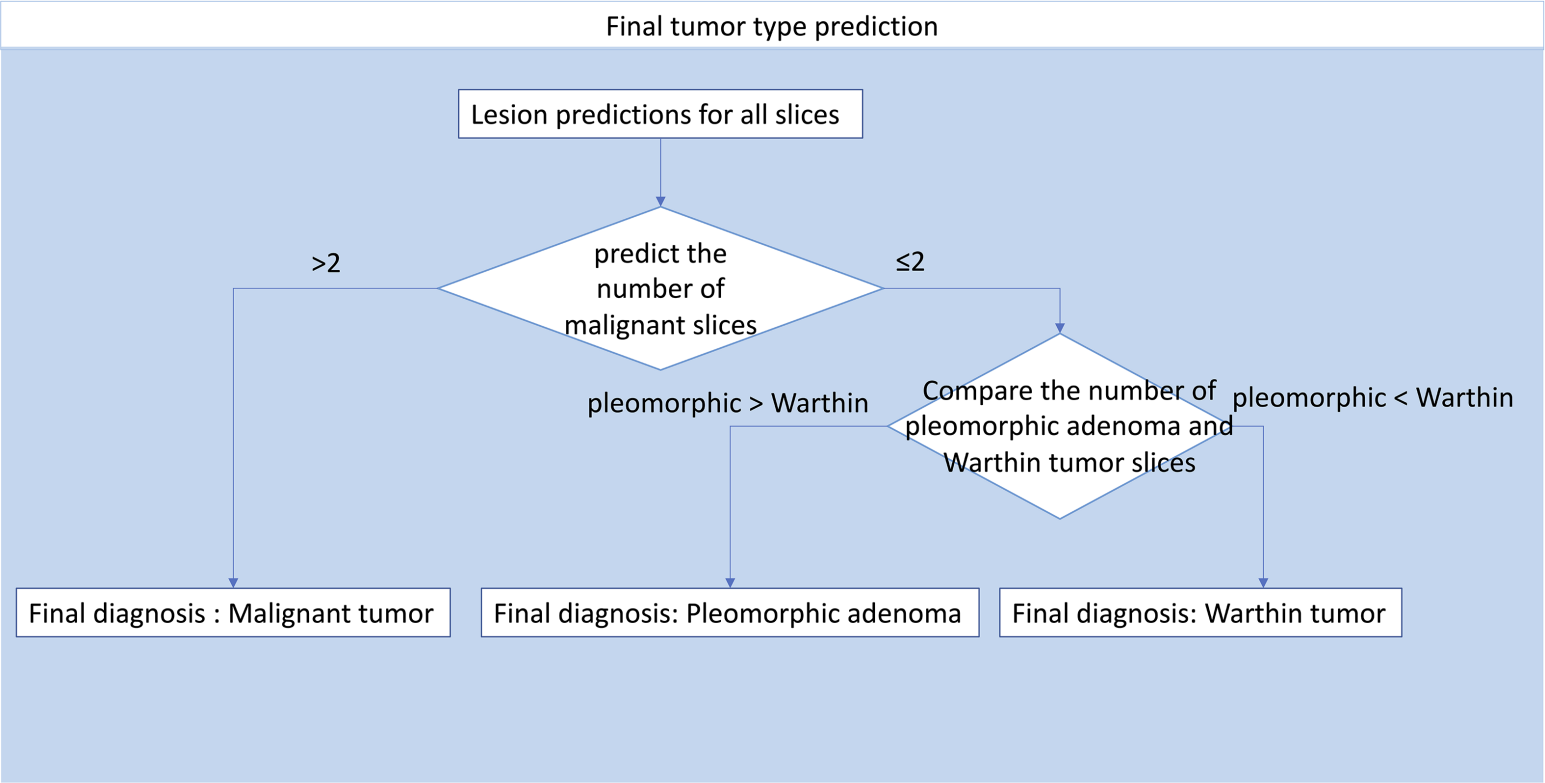
Workflow integrating all slices to predict the final diagnosis.

The slice probability was acquired by model prediction. To obtain patient diagnoses, a decision-making process was developed (**Figure 5**). The decision-making process was as follows: 1. Patients with more than two malignant slices were diagnosed as having a malignant tumor, and patients with no more than two malignant slices were diagnosed as having a benign tumor; 2. For patients with benign tumors, the tumor type was decided by slice number comparison. If the number of pleomorphic adenoma slices was greater than the number of Warthin tumor slices, the patient was diagnosed with pleomorphic adenoma, and vice versa.

## Results

**Figure 6:**
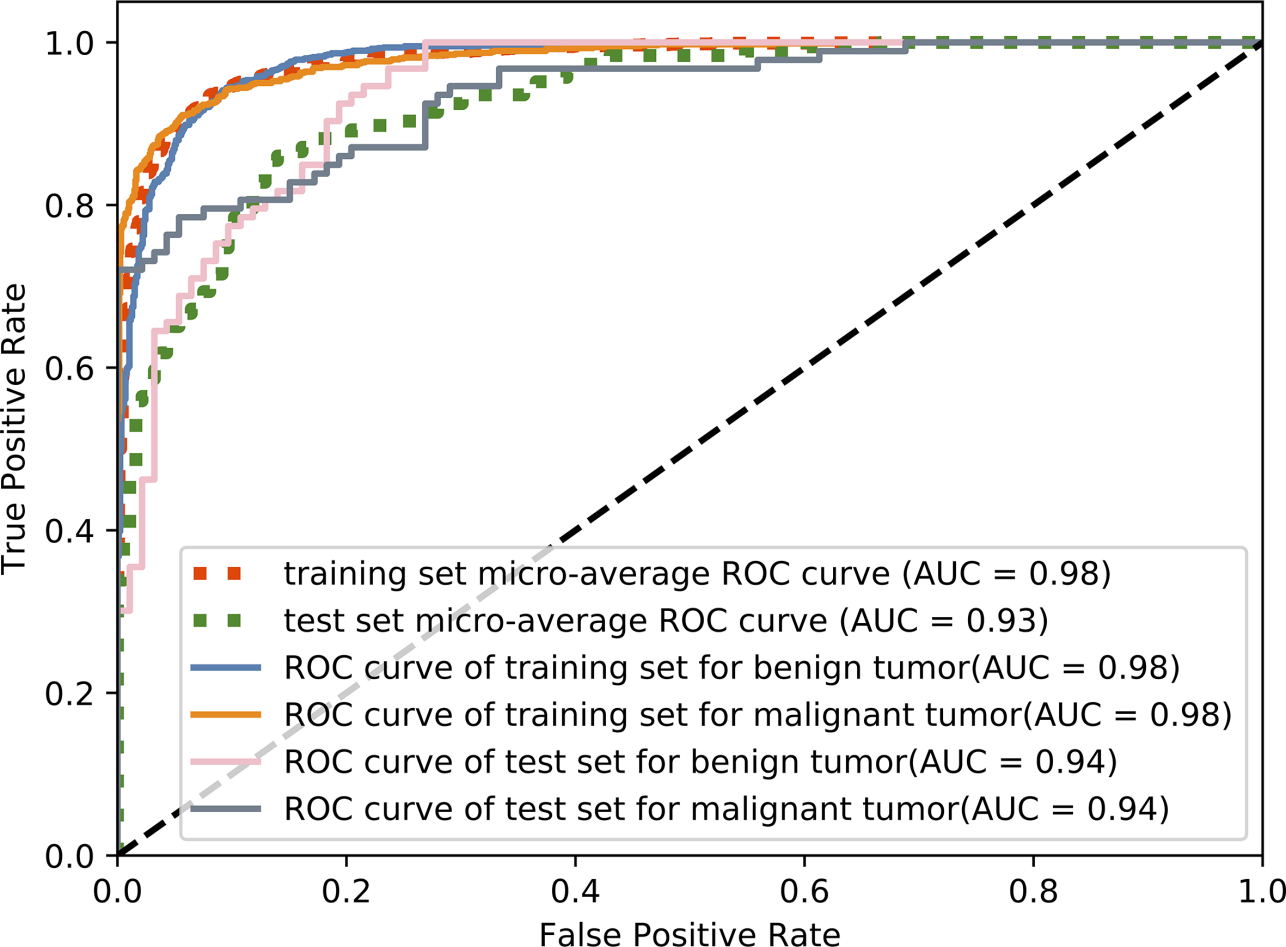
The ROC curves for predicting different classes of tumors using our proposed method.

The accuracy of slice classification was 92.94% for the training set and 82.18% for the test set. **Figure 6** shows that the area under the micro-average ROC curve (micro-AUC) was 0.98 for the training set and 0.93 for the test set. **Table 3** shows results of different training strategy for the single MR slice. For (a-c), the model was trained with single-modality image series. Pairs of MR series were integrated into the different image channels for training the model in (d-f), and all three MR series were used for the image channels in (g). The accuracy of the model when trained using single-modality image series in the channels was 0.706 (T1-weighted), 0.739 (CE-T1-weighted), and 0.707 (T2-weighted). Using pairs of image series boost the accuracy (**Table 3**). The use of all three image series (T1-weighted, CE-T1-weighted, T2-weighted) and the parotid gland contour images in the channels yields the best performance.

**Table 3:** Comparison of the accuracy for different channel compositions in asingle MR slice.

	Input image modality	accuracy
(a)	T1-weighted	0.706
(b)	CE-T1-weighted	0.739
(c)	T2-weighted	0.707
(d)	T1-weighted, CE-T1-weighted	0.702
(e)	T1-weighted, T2-weighted	0.798
(f)	CE-T1-weighted, T2-weighted	0.776
(g)	T1-weighted, CE-T1-weighted, T2-weighted (proposed)	0.822

(a), (b), and (c) use only a single series to train the model; (d), (e), and (f) use two types of MRI series for training; finally, the model (g) was trained by the proposed method using all three MRI series in the image channels.

Details of the results for the single MR slice prediction are shown in **Table 4**. Our model has good performance in distinguishing images that did and did not contain lesions and achieves an accuracy of 0.882 in identifying benign from malignant tumors. The accuracy in distinguishing pleomorphic adenoma and Warthin tumors among benign tumors was 63.4%.

**Table 4:** Parotid gland tumor classification results for different types of tumors in a single MR slice.

Types of tumor	Accuracy [95% CI]	Sensitivity [95% CI]	Specificity [95% CI]
Benign vs malignant	0.882 [0.827, 0.921]	0.946 [0.873, 0.980]	0.817 [0.721,0.887]
Pleomorphic vs Warthin tumor	0.634 [0.533, 0.725]	0.695 [0.560,0.805]	0.529 [0.354,0.698]
Without lesion vs with lesion	1 [0.986, 1]	1 [0.948,1]	1 [0.975,1]

**Figure 7:**
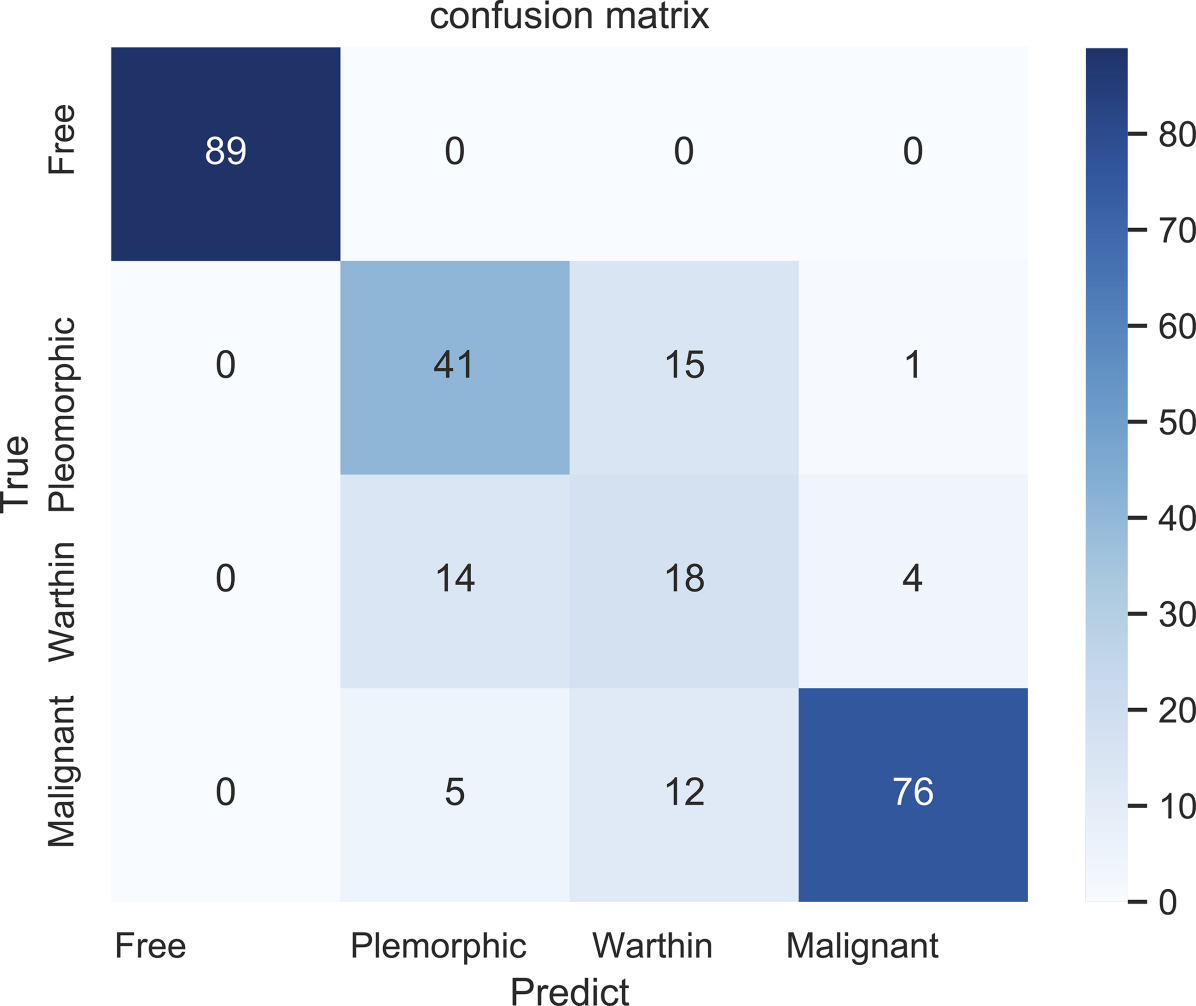
The confusion matrix for the four classifications: free (no tumor), pleomorphic adenoma, Warthin tumor, and malignant tumor.

**Figure 7** shows the confusion matrix of our proposed prediction model for individual slices. Each element (*x*, *y*) in the confusion matrix represents the number of samples with true class *x* that was classified as being in class *y*. The overall accuracy was 81.45%. The accuracy for the first class was perfect, as all 89 cases were correctly classified. In identifying benign tumor cases, sixteen cases (28%) and eighteen cases (50%) of pleomorphic adenomas and Warthin tumors, respectively, were misclassified. The accuracy in predicting malignant tumors was 81.72%.

**Table 5** shows the results from diagnosing twenty-one patients in the test set (8 with pleomorphic adenomas, 5 with Warthin tumors, and 8 with malignant tumors) by using the decision tree method (**Figure 5**). Six (75%) and three patients (60%) were correctly classified as pleomorphic adenoma and Warthin tumor, respectively. Only one patient in the benign tumor class was misidentified, and all eight patients with malignant tumors were correctly predicted.

**Table 5:** Accuracy of the decision-tree script in performing integrated prediction with the test set.

Tumor type	Number of patients	Number of correctly predicted patients
Pleomorphic adenoma	8	6
Warthin tumor	5	3
Benign tumor	13	12
Malignant tumor	8	8

## Discussion

In this study, we developed a model to predict the type of parotid gland tumor. The results show that our model can identify the type of parotid gland tumor at the slice level. Experiments show that using multichannel images as the input can improve the model’s ability to identify tumor features. The model can thus assist doctors in quickly determining tumor classifications in clinical practice. Using artificial intelligence modeling methods, the accuracy in predicting benign and malignant parotid tumors can be further improved, the prognosis can be evaluated, and a reasonable diagnosis and treatment plan can be formulated for patients.

Pathological analysis takes time and is expensive, resulting in a heavy financial and psychological burden for the patient (33). The main advantage of adopting deep learning into the prediction of the type of parotid gland tumor is that the deep-learning method can inform the physicist which patient may have a tumor or cancer faster than pathological analysis (34). With the use of deep-learning models, the patient’s condition could be narrowed and locked into benign or malignant type. This method may be useful for improving the efficiency of routine clinical practice and saving time in patient treatment.

In MR image preprocessing, due to the limited size of the image itself, we compared the performance between the multichannel images and single-channel images as the network input. Each dataset was trained with the same strategy, and the final average accuracy was 71.7% for single-channel input and 75.8% for double-channel input, which were 10.5% and 6.4% lower, respectively, than our proposed method. The neural network can extract features from different MR series, so we hypothesize that the use of multiple channels may boost model performance in diagnosing the type of parotid gland tumor; the obtained experimental results show that the performance of the model is effective. From **Table 3**, as the number of channels and integrated MR series increases, the accuracy of the model also gradually increases.

In training the model, we chose the current prevalent ResNet18 network as the backbone. The residual blocks prevent disappearance of the gradient to minimize the effects of the degradation problem after many iterations (35, 36). In our practice, we made some modifications to ResNet18 to adapt it to our classification task.

It should be noted that the segmentation of the tumor and parotid gland had to be performed manually by a physician, and the last prediction step involved a simple decision tree. In the future, these steps could also be performed by the deep learning-based model (autosegmentation of the tumor and parotid gland ROIs and integration of all patient slices to predict the tumor type by using a neural network).

The final model showed good performance in predicting pleomorphic adenoma and Warthin tumors. The prediction result for Warthin tumors seemed to be worse than that of the other classes, as fourteen cases (77.8%) of Warthin tumors were misidentified as being pleomorphic adenomas. We consider two reasons for this. First, our dataset was uneven, as the number of Warthin tumors was too low; therefore, the model performance in distinguishing pleomorphic adenomas and Warthin tumors was worse than that in identifying benign from malignant tumors. Second, benign tumors may share certain features that makes it difficult to distinguish the two types. In the future, a specific model for predicting different benign tumor types will be generated that may outperform the current model. Consequently, here we proposed a script that could can accurately distinguish between benign and malignant tumors.

There were some limitations in this study. First, we did not validate our model with an external dataset, which could have been valuable in demonstrating the reliable performance of the model. Second, we combined three types of MRI series (T1W, T2W, CE-T1W). During routine diagnostics, some series may not be acquired. Third, we proved the feasibility of using multiple channels to predict the type of tumor; however, the performance between benign tumors was not sufficiently precise. Furthermore, our data did not include examples of three other classes of parotid gland tumors. Given the lack of data on these other three classes of tumor, this study merely explored the feasibility of the above methods for the three classes with sufficient data.

In the future, larger-scale, multicenter studies are required for full validation of the model. We will enroll more patients in our study to train the model for diagnosing all six classes of parotid gland cancer.

## Conclusion

In this study, we proposed a novel method combining clinical experience and a deep-learning method to diagnose parotid gland tumors. We proved the feasibility of the method, trained the model for predicting tumor types, and developed a script to analyze the final prediction. We propose that the results of this study will help physicians diagnose tumor types in patients faster. It can improve the effectiveness of routine clinical practice for these tumors. In the future, this model could be used to assist young doctors in preventing misdiagnoses and other mistakes that could be made from working long hours.

## Data Availability Statement

The datasets presented in this article are not readily available because the data are not publicly available due to privacy restrictions. Requests to access the datasets should be directed to BF, 411593150@qq.com.

## Ethics Statement

The studies involving human participants were reviewed and approved by ethics committee, Fudan University Shanghai Cancer Center.

## Author Contributions

BF and XX: drafting the manuscript. BF: processing the data, developing the deep learning model and script. XX, QH, YY, and YM: acquisition of data and generated research ideas. JW and WH: provided guidance on methodology and overall project, and reviewed manuscript. LS: provided lab and technical support. All authors contributed to the article and approved the submitted version.

## Funding

The work was supported by the Zhejiang Provincial Health Science and Technology Project. Grant number: 2021KY396.

## Conflict of Interest

The authors declare that the research was conducted in the absence of any commercial or financial relationships that could be construed as a potential conflict of interest.

## Abbreviations

CAD, computer-aided diagnosis; FNAC, Fine-needle aspiration cytology; T1W, T1-weighted; CE-T1W, contrast-enhanced T1-weighted; T2W, T2-weighted; TR, Repetition Time; TE, Echo Time.
